# Language, Childhood, and Fire: How We Learned to Love Sharing Stories

**DOI:** 10.3389/fpsyg.2021.787203

**Published:** 2022-01-27

**Authors:** Gerhard Lauer

**Affiliations:** Gutenberg Institute of World Literature and Written Media, Johannes Gutenberg University of Mainz, Mainz, Germany

**Keywords:** literary anthropology, evolution of language, evolution of storytelling, evolution of childhood, taming of fire

## Abstract

Stories do not fossilize. Thus, exploring tales shared during prehistory, the longest part of human history inevitably becomes speculative. Nevertheless, various attempts have been made to find a more scientifically valid way into our deep human past of storytelling. Following the social brain hypothesis, we suggest including into the theory of human storytelling more fine-grained and evidence-based findings (from archaeology, the cognitive sciences, and evolutionary psychology) about the manifold exaptation and adaptation, genetic changes, and phenotypic plasticity in the deep human past, which all shaped the emergence of storytelling in hominins. We identify three preconditions for humans sharing stories: first, the long evolution of language in the different taxa as one of the preconditions of ostensive signaling; second, the pivotal role of childhood in the evolution of collaborative intentionality; and third, the role of fireside chats in the rise of elaborative (i.e., narrative) sharing of stories. We propose that humans, albeit perhaps no other hominins learned to understand others through sharing stories, not only as intentional agents, but also as mental ones.

## Anthropologists Unite!

The longest part of the human history of storytelling is uncharted territory. This is so because neither stories nor sharing them fossilize in a world without writing. Exploring the tales shared during deep human history will therefore remain speculative (Turner and Maryanski, 2015). Nevertheless, literary anthropology needs to find a scientifically valid way into our deep past of storytelling. Insights have emerged from various fields, such as archaeology findings and ethnographic fieldwork, evolutionary and developmental psychology, and the cognitive sciences (e.g., Dunbar et al., 2014). To turn speculation into plausible explanation, literary anthropology needs to integrate the disparate data, models, and theoretical concepts of this wide variety of disciplines (Carroll, 2020). While integration provides an important empirical lens for examining storytelling, anthropological research remains largely divorced from the body of theoretical work on culture and evolution within the humanities in general, and social anthropology, in particular. It is rare that anthropologists bridged the divide between evolutionary and social anthropology (Eriksen, 2006: 23). They include Barnard (2011, 2012) or more recently Wengrow and Graeber (2021). Kupers and Marks (2011) appeal—“Anthropologists unite!”—remains valid, because the opposing views in anthropology hamper the efforts of literary anthropology to understand both the symbolic and the evolutionary nature of human storytelling as part of a single framework.

Despite the fundamental discord in research on human origins, literary anthropology has recently established some promising avenues for illumining how humankind began to share stories. The concept of the “social brain” encompasses most of these attempts. It represents a major shift in efforts to understand the human history, at least since around the Middle Pleistocene in the light of a fundamental tendency toward prosocial behavior (Watts et al., 2016). Understanding the importance of sharing stories is part of this larger shift in theorizing the evolution of human prosocial behavior, which enabled hominins to better adapt to increasingly diverse habitats across the globe (Carroll, 2015). Based on the social brain hypothesis, we conceptualize storytelling as a specific form of intentionality, namely, collective intentionality, which enabled humans to develop complex cultural practices and concepts (Tomasello and Rakoczy, 2003). In the evolution of social cognition, which is unique to the modern human species, narrative practice was central to individuals and groups developing collective cultural products (Gallagher and Hutto, 2008). Stories are one very effective way of modeling the world on the level of collective behavior because stories teach hominins to understand that others have thoughts and values, expectations, desires, and beliefs that are worth learning from. Hence, anatomically modern humans model other humans as mental agents, not only as intentional agents.

Yet proponents of the social brain hypothesis are divided over the precise adaptive function of sharing stories. While some have claimed that humans began to enjoy sharing stories, because they directly affect the survival, others have argued that sharing stories is a by-product (i.e., impacts survival merely indirectly or as exaptation). In this regard, the debate on Pinker (1997) cheesecake hypothesis, that a preference for the arts is but a non-adaptive exploitation of adaptive sources of pleasure, is central (Carroll, 1998). So, too, is Pinker’s critique of the theory of the “literary animal” (Gottschall and Wilson, 2005; Pinker, 2007), which, in contrast, posits that storytelling has a direct adaptive benefit. As critics have stressed (Mellmann, 2012), this debate—adaptation versus by-product—limits research on the sexual selection, and about how mating schema, confidence tricksters, or bride abduction influence narrative plots to this day and have triggered aesthetic pleasure for thousands of years. The often-used metaphor of the literary animal is misleading. Even worse, it tends to reduce the debate to the notion that stories are mostly about “humans facing problems and trying to overcome them” (Gottschall, 2013). Although we have merely outlined the debate (concentrating on the period 1995–2015), it is evident that literary anthropology insists on exploring the adaptive benefits of story sharing rather than seeking to understand the enabling conditions.

Instead of extending the adaptation versus by-product debate, we highlight three evolutionary developments in the rise of storytelling: the emergence of a vocal and grammatically complex language, the evolution of childhood, and the taming of the fire (which expanded language to narrativity). During hominid evolution, language became more than signaling, namely, a tool for ostensive signaling (Dissanayake, 2000). Such narrativization gradually enabled hominins to simulate perspective of another person and to relate simulation to their own perspective (Harris, 1996). Childhood and the taming of fire both fostered this narrativization.

These three evolutionary steps remind us that other developments, such as foraging or weather shamanism, as well as climate or demography, also need to be considered in modeling the deep history of storytelling. We argue that the three developments—language, childhood, and fire—directly influenced the origins of human narration, even if they did not drive human evolution alone. But language, childhood, and taming fire are gradual phenomena involving multiple stages. Thus, tracing the long evolutionary history of humankind is more important to understanding the specificity of the imaginative culture of humans than searching for a proximate adaptive function. Emerging from the convergence of various ancestral hominin traits, storytelling coevolved into a typically human cooperation strategy. Over time, individual narrative styles (Wobst, 1977; Wiessner, 1984) and other ostensive signaling became increasingly important. In line with Mithen (1996); Dissanayake (2000), and Smith et al. (2017), we understand that the sharing of stories as a means of conveying information about others as mental agents, and about their social identity, to assess the individual behavior in complex foraging bands and their social norms. Further, we suggest that humans, though perhaps not other hominins have learned to understand others as mental agents.

## Vocal Language, Not Solely a Human Privilege

Recent findings in genetics suggest that about 300,000 years ago, many human lineages coexisted and sometimes interbred, including the anatomically modern human (Hublin et al., 2017). This time span almost doubles the realm of human evolution and enabled our lineage to glean from the speech production apparatus of our sister lineages. Archaeological records have provided rich insights into Neanderthal culture: the complexity of its fiber, leather, and clothing technology (Wales, 2012; Hardy et al., 2020); its ample medical knowledge, among others, about the use of bitter herbs or about dental treatment (Hardy et al., 2012); or about making fires using manganese dioxide (Heyes et al., 2016). These findings narrow the gap between the now extinct lineages and anatomically modern humans. Further evidence, albeit still circumstantial, includes the burial of a Neanderthal child (Balzeau et al., 2020), or ritual cannibalism, and secondary burials (Frayer et al., 2020). More robust evidence, on decorative bones, feathers, or constructions (Jaubert et al., 2016; Majkić et al., 2017; Finlayson, 2019), enables attributing complex social and cultural life to our sister lineages. Evidence in support of this attribution includes anatomical findings about the anatomy and physiology of the vocal tract, and about breathing control and acoustic sensitivity (Conde-Valverde et al., 2021), which indicates how close Neanderthal cochlear volume and audition (Beals et al., 2016; Stoessel et al., 2016) were to modern humans. Together, this mounting evidence demonstrates that symbolic behavior and speech also existed beyond our lineage.

The available data do not allow drawing direct inferences on the prehistoric linguistic structure. That, in fact, is impossible. Nevertheless, anatomical data dating back to approximately half a million years, in particular about the vocal and auditory apparatus in hominin lineages, and about the Neanderthal symbolism and composite tool building, suggest that language as a vocal system, which is characterized by sounds mapping meaning and by recursive grammar, gradually developed from proto-languages (Mithen, 2005) in hominin evolution. A growing body of evidence suggests that language might not be exclusively linked to the last surviving hominins: us (Dediu and Levinson, 2013, 2018). By no stretch of the imagination did the Neanderthals, and perhaps even other hominins, have a large lexicon and propositional encoding. Yet, they managed to culturally adapt to different climates, from the Arctic to the Mediterranean, to bury their dead with mortuary ceremonies, or to penetrate deep caves—even if their lexicon, habitats, and cultural practices seem less diverse than those of modern humans. One indication of the gradual differences between the Neanderthals and modern humans is the proliferation of ornaments in the Upper Paleolithic. This array coincides with the reach of modern humans within Eurasia and also with the eventual disappearance of the Neanderthals (Kuhn et al., 2001).

In this perspective, language, as a gradual phenomenon, evolved in multiple steps. The question, however, is: what might these major steps be in the evolution of human language? Did language originate in a more holistic protolanguage, which subsequently evolved toward segmented and composed language structures, as Arbib (2005) and Mithen (2005) have suggested? Such a holophrastic protolanguage implies the existence of multipropositional utterances, where complex processes, such as burial rituals or composite tool making are compressed into one or a few unitary utterances. Still, as critics have pointed out (Tallermann, 2008), such a protolanguage needs to involve a holistic cognitive mode, an inevitably complex mode featuring highly sophisticated conceptual planning capacities, large mental storage, and fast lexical retrieval vastly superior to those available to *sapiens*. However, such a protolanguage consisted of components, which due to greater mental fluidity and conscious learning (Cleeremans et al., 2019), became increasingly hierarchically organized and recursively structured—if not on the level of syntax, then on the level of semantics and pragmatics, and including ever greater contextual inferences. There is no need for a rigid phrase structure on the surface level since even today’s languages differ widely on that level (Austin and Bresnan, 1996).

The gradual evolution of language over at least 500,000 years in hominin lineages is one of the major stepping stones in the evolution of storytelling. This claim is supported by increasingly better, although not conclusive evidence from linguistics and cognitive psychology, from genetics and archaeological sites. In this perspective (i.e., a long evolution of diverse lineages), hominins are very articulate beings. Their language skills enabled them to learn complex behavior, such as making clothing or using ornaments to adorn the body with paint, beads, and bird feathers (Rodríguez-Hidalgo et al., 2019). These observations suggest that the Neanderthals and other hominins already used language to learn how to make composite tools or to signal the meaning of personal ornaments. They were capable of receiving information from others, communicatively, such that utterances may have been a focal point. This marked a shift away from the narrator’s situation (Mellmann, 2010, 2014), and thus enabled the Neanderthals and other hominins to simulate a specific perspective on processes and phenomena (Cosmides and Toby, 2001).

We nevertheless need to discuss whether Middle Paleolithic symbolism differs in complexity from anatomically modern humans (Chase and Dibble, 1987). We suggest that the difference lies not in language itself, but in the extent of its narrativization. Almost no non-utilitarian composition has survived for *Homo erectus*, whereas symbolic behavior is well documented for the Neanderthals (Prévost et al., 2021). However, comparing the Neanderthals and anatomically modern humans, the degree of including other perspectives and values, beliefs and expectations might still be assumed to differ from the narratively more complex stories shared between *sapiens*. Even if the Neanderthals had shared meanings, and even if these were consciously learned and construed, while inducing thinking, action, and follow-up communication between individuals and the band (Henshilwood, 2014), differences may still have existed between sister lineages. In this sense, hominins knew that individual and ostensive signaling preceded referential information (Sperber and Wilson, 1986), yet to a different degree. Their ability to organize language along narrative practices varied, and therefore the complexity of their collective intentionality (Hutto, 2008). We suggest that ostensive signaling varies between sapiens and their sister lineages to the extent that task-relevant information is available to all co-actors (Vesper et al., 2021). Conventions, norms, and beliefs are not directly available to co-actors and need to be expressed verbally if not narratively. We assume that the ability of telling stories about norms and beliefs differs between hominin lineages. The degree of narrativity, and the extent to which norms and beliefs are narrated, is closely related to how far they understood intentionality of others and how far hominin lineages could scale up collective intentionality to group life. Groups become more interdependent and build a stronger group-mindedness by the growing ability to share knowledge about conventions, norms, and beliefs (Tomasello et al., 2012). The degree to which *sapiens* but no other hominins can mark individuals as members of a particular cultural group is shaped by the ability of humans to narrativize information into stories. If the cultural and biological evolution of *Homo sapie*ns in Africa occurred as a mosaic of local developments (Scerri et al., 2018; Sehasseh et al., 2021), language might be assumed to have evolved from a signaling tool into the hominin ability to understand intentions of others and further into understanding others as mental agents holding complex beliefs and norms.

## Childhood Revisited

In the course of evolution, hominins became better learners mainly by integrating the knowledge of others. A tendency toward natural pedagogy (Gergely and Csibra, 2006) makes hominid evolution unique. Ostensive signaling is central to learning. It involves providing recipients with cues that they should devote their cognitive resources to figure out the content of messages. Learning, however, is risky and costly in evolutionary terms. Therefore, learning is invested solely to the extent that children receive sufficient nutritional and protective support from their parents, as claimed by the developmental support hypothesis (Snell-Rood and Snell-Rood, 2020). Hominins became voracious learners. They adapted to a wide range of habitats through this kind of support, which today we call as childhood. Childhood increases brain size and delays maturation. Childhood and youth evolved significantly during hominid evolution (Bogin, 1990), though not simply in one direction. In analyzing tooth calcification, Smith et al. (2010) and Smith (2013) found that the Neanderthals may have reversed the trend toward prolonged childhood. The characteristically prolonged development of modern humans might have evolved fully after these taxa diverged. In addition, it might explain why no hard evidence has thus far been found for Neanderthal rock art (Pons-Branchu et al., 2020). Although the lineages are close, differences still exist in the long history of hominin taxa. This also holds true for storytelling.

It is difficult to establish how much Neanderthal tales differ from those of *sapiens*. One difference, we suspect, is the role of skilled narrators in more complex forms of audience-narrator cooperation. Indirect support for this claim comes from the evolution of childhood, which seemingly occurred over millions of years, as suggested by data on the infant-mother ratio and on the birthing of relatively large neonates since the *Ardipithecus* (DeSilva, 2011). Mutualistic collaboration, as evident in sharing stories, needs childhood, because a prolonged childhood enables humans to learn to cooperate with the group-oriented and coordinated individual behavior, as captured best by Tomasello’s (2009) concept of collective intentionality. Yet this line of research has been overlooked how raising children contributes to the evolution of collective intentionality. Childcare is *the* place for developing collective intentionality, perhaps even more than hunting. In particular Sarah Hrdy (2009), based on ethnographic studies of today’s forager societies, has impressively discussed the decisive role of parenting in the evolution of collective intentionality. Caring is far more than maternal care: it involves highly coordinated behavior, such as mother and pair-bonded fathers, siblings, unrelated alloparents, and post-menopausal grandmothers (Hawkes, 2014). Over time, such collaborative breeding distinguished us from our sister lineages and contrasted ever more sharply with our closest primate relatives.

Alloparenting or allocare, that is, cooperative breeding in the extended family, drove the evolution of human primates (Konner, 2010: 426–451). Distinguishing joint and shared attention (Schweikard and Schmid, 2020) suggests that prolonged childhood is the principal environment for learning the differences between many agents around children and for developing a basic consensus on how alloparents cooperatively raise a child (joint attention). Parenting agents intend to achieve a collective goal, which includes the coordinating knowledge between many parents. This requires understanding others not only as intentional agents but also as agents with shared habits, norms, and beliefs (Tomasello and Rakoczy, 2003). While children learn to incorporate the preferences of others, the children of *sapiens* seem to have learned better than any other hominin to understand others as mental agents.

In this interplay of different roles, the cooperative skills of infants as well as those of adults have improved and transformed infancy, childhood, and adolescence over time toward more complex cultural learning (Tomasello and Gonzalez-Cabrera, 2017). Learning in infancy requires taking perspective and treating others as intentional agents. Thus, it involves learning that others do things on purpose—and due to their social norms or habits. This makes it the most probable foundation for all subsequent, more advanced forms of social cognition (including storytelling). Allocare requires knowing what others know, as well as recognizing the differences between them and their roles, and communicating what others need to know to raise helpless children (shared intention). It is difficult to imagine that alloparenting works without language to share the information about actions and roles over nearly two decades (collective acceptance). Finally, the evolution of neoteny depends heavily on collective emotions to know what matters for child and alloparents, and what needs to be done next (collective emotions). In sum, allocare formed a large, often the largest part of anatomically modern human life in the Pleistocene (Gopnik et al., 2020). We learned collective intentionality in childhood. As we became children, we became humans.

Early-life experiences shape later cognitive abilities more than the later life experiences. This applies not only exclusively to human evolution. But it holds true particularly for the divergence from other taxa of anatomically modern humans, as their childhood lasts longest, that is, children remain connected with male and female allomothers for the longest period. Hrdy calls this the cooperative breeding hypothesis (Hrdy, 2009, 2016). Cooperative breeding is closely related to the vocal flexibility: “Once vocal control has evolved to help infants secure care, it is only a small step to producing utterances in context-specific ways.” Further: “[This] may only be possible against a background of other psychological skills, such as the ability to share intentions and attention, and well-developed comprehension” (Zuberbühler, 2012: 80). Language is highly useful, not only between alloparents but also for children, for instance, to attract carers. A child shapes its calls into babbling and widens its vocalization and its voluntary control of vocalization (Goldstein et al., 2003; Burkart et al., 2018). Changes in actions are reflected by the changes in vocalization. Both caregivers and children learn to coordinate vocalization in the shared tasks. To effectively communicate and collaborate, both sides must develop a shared understanding of each other’s intentions (Trevarthen, 1979; Murray and Trevarthen, 1986; Moll et al., 2021) and a growing sensitivity for the differences in vocalization. To this day, motherese is not a holistic, non-phrasal language but rather a language with strong pragmatics, highly controlled prosody, and a rapidly complex and recursive syntax.

Moreover, child-directed speech is not symmetrical. It has different roles and playing with roles is part of things. Knowing whom to learn from is fundamental for everyone involved in this long history of childhood communication. Gradually, the evolution of roles included more members of bands than merely parents. Data on the average territory of small-scale hunter-gatherer bands suggest that behaviorally modern humans have more elaborate roles than their close relatives, because transportation distances and networks increased compared with Neanderthal times (Marwick, 2003; Nash et al., 2013).

This asymmetry becomes important for sharing stories among *sapiens*. Childhood changes the use of language, from purely factual use, with the speaker as the deictic center of communication, to narratives, which grant narrators the freedom to transcend the here and now. A kind of division of labor, between more skilled narrators and more experienced, elderly persons sharing their knowledge with younger and less experienced ones, is a default constellation of the storytelling that emerges in alloparental care. Against the romantic notion that mythical stories are the core of storytelling, we follow Scalise Sugiyama (1996) in emphasizing how strongly forager societies depend on reliable knowledge about their lifeworld. Storytelling is a part of natural pedagogy and exhibits the typical signs of ostensive communication (i.e., eye contact, exaggerated prosody, and gestures). It is part of motherese (just listen to how we explain new things to novices).

Sampling data from over 50 forager cultures on five continents and including over 30 language families, Scalise Sugiyama (2021) found that storytelling predominantly serves to transmit generalizable knowledge rather than mythical tales. Although mythological stories have been found in hunter-gatherer bands, knowledge about places and weather, animals and plants, as well as about social rules, such as stories about bullies and tricksters (as shown in refs. Boyd et al., 2011; Nakawake and Sato, 2019), dominate the stories shared in hunter-gatherer cultures as recorded since the nineteenth century.

In the course of evolution, storytelling largely became an expert practice, at least in *sapiens*. Storytellers adapted their repertoire to their audience, looked directly at their listeners, and spoke in a different voice than in everyday exchanges. Often, they rhythmically accompanied their storytelling with singing, vocal mimicry of animals, imitated the actions of the small number of characters in their stories, used repetitions, direct address, laughter, or inserted songs during their performance (Scalise Sugiyama, 2017). Narrators—from mothers to experts—narrativize language. Overwhelmingly, their stories do not feature mythical heroes (Biesele, 1993), but characters which make sense in egalitarian cultures, where people depend on one another rather than on dominant figures (Boehm, 1999). Narrators therefore tell stories about cooperation, about equality between the sexes, and about egalitarianism. Stories become tales about social norms, including the role of storytellers. Good storytellers cooperate with their audience and, in return, become preferred social partners with greater reproductive success (Smith et al., 2017). Once more, this points to the selection pressure under which the socially more cooperative find themselves.

## Fireplace Talks

Humans started to love sharing stories under the selection pressure exerted by a more social brain. Yet storytelling behavior was driven by several factors. The taming of fire is a third major factor in any broader account of the evolution of human storytelling. Recent research has found evidence for a deep history of the controlled use of fire by hominins. Fire became embedded in the hominid behavior approximately 1.5 million years ago, although artifactual evidence for early hominin use is still rare, which suggests that fire was used merely sporadically. From the Middle Pleistocene, hominins deliberately made fires, with hearths becoming more common in the last 400,000 years (James, 1989; Dunbar and Gowlett, 2014). In addition, this affected the storytelling. Hominins used fire at night and in caves (Gowlett, 2016), two very special environments for hominins. For example, Bruniquel Cave, where hominins would meet about 350 m from the entrance (Jaubert et al., 2016). While fire makes nights and caves special, we have no record of what they meant for those who ventured into dark caverns. Ethnographic records of today’s hunter-gatherers help to understand what might have happened at those special places in hominid times. As Wiessner (2014) has observed, fireside talk among Ju/’hoan (!Kung) bushmen telling stories at night differs from their daytime exchanges. While everyday information and gossip dominate during daylight (Dunbar, 2004), and at night, long tales about traveling through the time and space, about humans metamorphosizing into animals, and animals into humans, about cosmic phenomena, such as the moon and the stars or ancestors, about ghost and demons, structure the stories shared by firesides. In a number of societies, it is forbidden to tell tales during daytime (Scalise Sugiyama, 2017). Narrative tales belong to the night and caves. They are more complex than the instructive stories shared during daytime.

The (narrative) stories told at firesides, in caves and at night, explain the larger scheme of human existence: why we die, what the world above and below us looks like (d’Huy, 2020a,b), why people go hungry, or why cheating is bad. While this cosmic world is not completely unlike the world known to the narrator and the audience, stories often center on people, places, and animals familiar to the group. As the term “myth” is potentially misleading, we speak of narrative tales. The rules in these stories are those of the band and concern reciprocity, marriage, and the exchange of goods. Tales, however, paint a bigger picture of the world, and as such stir the imagination more than the everyday gossip. The substrate of the imagination “deal[s] with problem points in living which must always have characterized the hunting-gathering adaptation, such as uncontrollable weather, difficulty in procuring game, danger from carnivore attacks, and correct relations with in-laws” (Biesele, 1993:13).

Now, we might object that the fireside storytelling makes everything special. However, it requires more cognitive effort than ordinary language. Besides, it may have contributed to the emergence of a theory of mind and to metacognitive fluidity on a scale where *sapiens* began understanding each other as mental agents. Humans learned through stories to foster social cooperation, that is, to teach each other social norms while also expressing their identities.

As storytelling is costly (Smith et al., 2017), as it means walking into dark caves and conjuring up the extraordinary, a broader account of human evolution (including the selection pressure toward the social brain) needs to consider the taming of fire. Sitting around a campfire instead of running away from heat makes such occasions unique while the stories told in such moments encourage hominins to walk into special places, such as deep caves. In this sense, storytelling is more than gossip, and as such closely tied to special places and times (Flesch, 2007). We do not know whether such elaborate storytelling existed in other taxa (e.g., the Neanderthals). Bruniquel Cave, however, whose annular structures of broken stalagmites date back 176,000 years, suggests that even the Neanderthals wandered hundreds of meters into the darkness, guided by torches, to build what we now call the first architecture in world history. We can imagine them telling stories as they moved ever further into darkness. Quite likely, anatomically modern humans are not the only storytellers on earth, even if they are perhaps the most skilled. Storytelling, then, evolved gradually, over the course of the long human journey toward cooperative behavior. It started long before us.

## Discussion

Based on recent findings in various fields (paleo-archaeology, ethnography of forager cultures, comparative anthropology and anatomy, and cognitive psychology), we have attempted to offer solid empirical insight into the origins of storytelling. This has involved conceptualizing the evolution of sharing stories as a gradual phenomenon as a part of a larger process: the evolution of the social brain. We have highlighted the deep evolution of language against the background of holistic protolanguages, the pivotal role of childhood, and fire as central to the transformation from telling to narrating. It seems that humans rather than their sister lineages turned language into narrative. Childhood and the taming of fire were pivotal to the narrativization of language.

We have argued that more detailed empirical research is needed in literary anthropology, as is already well-established in visual arts research (e.g., Straffon, 2014) or musicology (e.g., Grauer, 2015). It is tempting to interpret the results as representing a closed research agenda. Nothing could be further from the truth, because the three factors that we have highlighted are part of a more complex history of human cooperative behavior. While they serve as a proxy, these factors account neither for the important role of climate changes nor for moving in very different habitats. Nor do they address the evolution of the sexes, or the differences and intersections between gathering and hunting, the back and forth in evolution (which involves many ratchet effects, but also many losses, which prove hard to detect). Evolution is a convoluted process, whose ups and downs elude any detailed account. Yet in line with an extended evolutionary synthesis (Pigliucci and Müller, 2010; Laland et al., 2014), where organisms modify environments and environments shape organisms, where physical development influences the generation of variation and is transmitted extra-genetically, we might think of the evolution of human imaginative culture as a process, where language, childhood, and fire shape, how we learned to love storytelling.

## Author Contributions

The author confirms being the sole contributor of this work and has approved it for publication.

## Conflict of Interest

The author declares that the research was conducted in the absence of any commercial or financial relationships that could be construed as a potential conflict of interest.

## Publisher’s Note

All claims expressed in this article are solely those of the authors and do not necessarily represent those of their affiliated organizations, or those of the publisher, the editors and the reviewers. Any product that may be evaluated in this article, or claim that may be made by its manufacturer, is not guaranteed or endorsed by the publisher.
